# Cost-effectiveness analysis of computer aided detection for tuberculosis in Ghana: A decision-analytic modeling study

**DOI:** 10.1371/journal.pone.0356244

**Published:** 2026-08-27

**Authors:** Yaw Adusi-Poku, Jacob Solomon Idan, Aliyu Mohammed, Bernard Wadie Adu, Emmanuel Kweku Nakua, Ellis Owusu-Dabo

**Affiliations:** 1 Department of Epidemiology and Biostatistics, School of Public Health, KNUST, Kumasi, Ghana; 2 National Tuberculosis Control Programme, Ghana; Stellenbosch University, SOUTH AFRICA

## Abstract

**Background:**

The financial sustainability of universal molecular testing for tuberculosis (TB) remains a critical challenge in high-burden settings. In Ghana, the national program utilizes GeneXpert MTB/RIF for confirmation, yet the high unit cost necessitates efficient triage tools. This study evaluates the cost-effectiveness of Computer-Aided Detection (CAD) based triage compared to the standard of care in Ghana.

**Methods:**

This is a prospective cross-sectional diagnostic accuracy study, with an embedded decision-analytic economic evaluation, using primary data from 701 symptomatic outpatients across 23 facilities in Ghana. The cost-effectiveness analysis compared the vendor-recommended threshold of 51 with locally calibrated CAD scores of 56 (weighted Youden) and 68 (standard Youden). These were evaluated against the current Standard of Care (symptom screening and human-read chest X-ray). Health outcomes were measured in Quality-Adjusted Life Years (QALYs) lost, applying a severe morbidity penalty to missed TB cases (False Negatives) to account for prolonged suffering. The primary outcome was the Incremental Cost-Effectiveness Ratio (ICER). Analysis was conducted with R version 4.3.

**Results:**

The CAD-based triage at the weighted Youden threshold of 56 was identified as the optimal clinical strategy, being both more effective and less expensive than the current Standard of Care (SoC). The total health system pathway cost for the CAD-56 strategy and SoC was US$23,449.01 and US$33,239.09, respectively. Threshold of CAD-56 instead of the SoC saved the health system US$9,790.08 on 701 individuals screened and yielding an incremental health gain of 10.08 Quality-Adjusted Life Years (QALYs).

**Conclusion:**

Implementing locally calibrated CAD4TB version 7 at an optimized threshold of 56 as a gatekeeper for molecular testing would be a dominant economic strategy for TB control in Ghana. The findings provide a preliminary evidence for further assessment with nationwide data before national rollout of AI-based triage.

## Introduction

Tuberculosis (TB) remains one of the most significant infectious disease threats globally [1]. Despite substantial progress in treatment success, TB continues to be among the leading causes of mortality in the World Health Organization (WHO) African Region. It accounts for approximately a quarter of the global burden [1]. Ghana is no different with an incidence rate of 126 cases per 100,000 population [1]. The introduction of molecular WHO-recommended rapid diagnostic tests (mWRDs) such as GeneXpert MTB/RIF has revolutionized TB diagnosis with high sensitivity and simultaneous detection of rifampicin resistance [2,3]. However, the high unit cost of implementation serves as a primary barrier to universal molecular testing in resource-constrained settings [2,4]. In Ghana, the implementation cost of a single GeneXpert test is estimated at $43.90 per person screened with cartridges, personnel, and logistics cost inclusive [2]. This high expenditure necessitates a more efficient gatekeeping strategy to ensure that expensive molecular diagnostic resources are prioritized for those with the highest probability of disease [5].

To address the excess expenses of GeneXpert on false positives generated by the standard triaging screening, which consists of symptom screening with human-read digital chest radiographs (CXR), coupled with the shortage of skilled radiologists and high inter-reader variability [6], the WHO recommended the use of Computer-Aided Detection (CAD) software as an alternative to human interpretation of CXR for TB screening and triage in individuals aged 15 years and older [5,7,8]. The incorporation of this new screening tool into the existing algorithm for screening pulmonary TB is key. In Ghana, CAD4TB version 7 has been piloted using a vendor-recommended threshold of 51 [9]. The replacement of the traditional human-read CXR with a CAD-integrated algorithm must be considered vis-a-vis an appropriate CAD threshold that carefully balances diagnostic cost savings against the severe health penalties of missing true TB cases (False Negatives). In the long term, the sustainability of this incorporation must be assessed in terms of true cost-effectiveness, factoring in both financial expenditure and patient morbidity

A significant knowledge gap exists regarding the cost-effectiveness of implementing CAD in West Africa. While some studies in Ghana [10,11] Zambia and South Africa have documented the clinical accuracy of CAD [12,13], cost effectiveness analysis still lacks although essential for guiding the Government of Ghana's policy decisions to scale up the CAD4TB intervention. This study assesses the cost-effectiveness of various CAD-based triage strategies (vendor threshold of 51 vrs locally optimized thresholds of 56 and 68) compared to the current standard of care in Ghana, which entails symptom screening followed by human-read CXR triage before GeneXpert testing. Crucially, this decision-analytic model incorporates the health penalty of missed TB diagnoses, measuring outcomes in Quality-Adjusted Life Years (QALYs) lost. The aim is to identify an optimal threshold that minimizes both health system costs and patient suffering, providing a robust scientific basis for a sustainable, national-scale rollout of CAD4TB.

## Methodology

### Study design and setting

This study was a multi-facility, prospective cross-sectional diagnostic accuracy study conducted over a 12-month period from July 2022 to June 2023. The study was implemented across 23 public health facilities in seven regions of Ghana, purposively selected to represent the country’s three ecological zones (Coastal, Middle Belt, and Northern Savannah) and various facility tiers (tertiary, secondary, and primary). All sites were equipped with stationary digital X-ray units integrated with CAD4TB version 7 software (Delft Imaging, Netherlands) [9] and had on-site GeneXpert testing capacity.

### Sample size

The sample size was calculated based on the WHO CAD calibration sample size which was developed using the Buderer’s formula [14]. The calculation used a specificity and sensitivity of 70% with 5% precision and 95% confidence.


$$\begin{array}{c} n_{neg/pos}=\frac{Z^{\text{2}}\times S\times (\text{1}-S)}{d^{\text{2}}} \end{array}$$

$$\begin{array}{c} n_{neg/pos}=\frac{{\text{1}.\text{9}\text{6}}^{\text{2}}\times \text{0}.\text{7}\text{0}\times \text{0}.\text{3}\text{0}}{{\text{0}.\text{0}\text{5}}^{\text{2}}} \end{array}$$

$$\begin{array}{c} n_{neg/pos}=\frac{\text{3}.\text{8}\text{4}\text{1}\text{6}\times \text{0}.\text{2}\text{1}}{\text{0}.\text{0}\text{0}\text{2}\text{5}}=\text{3}\text{2}\text{2}.\text{6}\text{9} \end{array}$$

Rounding up, the sample size needed was 323 for GeneXpert-positive or GeneXpert-negative. Accounting for 10% TB prevalence among OPD attendees [15], the final sample required for this study was 680 with 340 GeneXpert-positive and 340 GeneXpert-negative participants.. However, over-enrollment was done to account for potential attrition and invalid test results, achieving a final sample of 701. The sample obtained from each study site and distributed proportionally perthe OPD attendance is shown in Table 1.

**Table 1:** Sample Distribution across Study Sites and GeneXpert Outcome.

Region	Facility	Annual OPD Attendance	Proportion Annual OPD Attendance	Total Sample	GeneXpert-positive	GeneXpert-negative
**Greater Accra**	Korle-Bu Teaching Hospital	395,387	41.5%	135	44	91
	Ridge Government Hospital	200,000	20.9%	68	22	46
	Tema General Hospital	100,000	10.5%	34	11	23
	Ga South Government Hospital	100,000	10.5%	34	11	23
	Achimota Government Hospital	96,833	10.2%	33	11	22
	Amasaman Government Hospital	43,732	4.6%	15	5	10
	Ashaiman Polyclinic	16,655	1.8%	6	2	4
**Greater Accra Total**				**325**	**105**	**220**
**Ashanti**	Komfo Anokye Teaching Hospital	300,000	59.4%	49	25	24
	Kumasi South Hospital	87,669	17.4%	14	8	6
	Obuasi Government Hospital	48,921	9.7%	8	4	4
	Nkawie Toase Government Hospital	37,426	7.4%	6	3	3
	Konongo Government Hospital	31,027	6.1%	6	2	4
**Ashanti Total**				**83**	**42**	**41**
**Bono**	Sampa Government Hospital	35,974	39.4%	10	6	4
	Dorma East District Hospital	29,299	32.1%	9	5	4
	Tain Government Hospital	26,014	28.5%	7	3	4
**Bono Total**				**26**	**14**	**12**
**Western**	Half Asini Government Hospital	37,376	35.4%	56	50	6
	Prestea Government Hospital	30,056	28.5%	45	40	5
	Wassa Akropong Government Hospital	22,003	20.8%	33	29	4
	Nana Hima Dekyi Government Hospital	16,223	15.3%	24	21	3
**Western Total**				**158**	**140**	**18**
**Upper East**	Bolga Regional Hospital	75,425	68.5%	25	22	3
	War Memorial Hospital	34,657	31.5%	12	7	5
**Upper East Total**				**37**	**29**	**8**
**Volta**	Akatsi Government Hospital	28,995	100%	55	0	55
**Volta Total**				**55**	**0**	**55**
**Eastern**	Asesewa Government Hospital	18,029	100%	17	12	5
**Eastern Total**				**17**	**12**	**5**
TOTAL		**2,237,783**	**100%**	**701**	**342**	**359**

### Participants and eligibility

**Fig 1:**
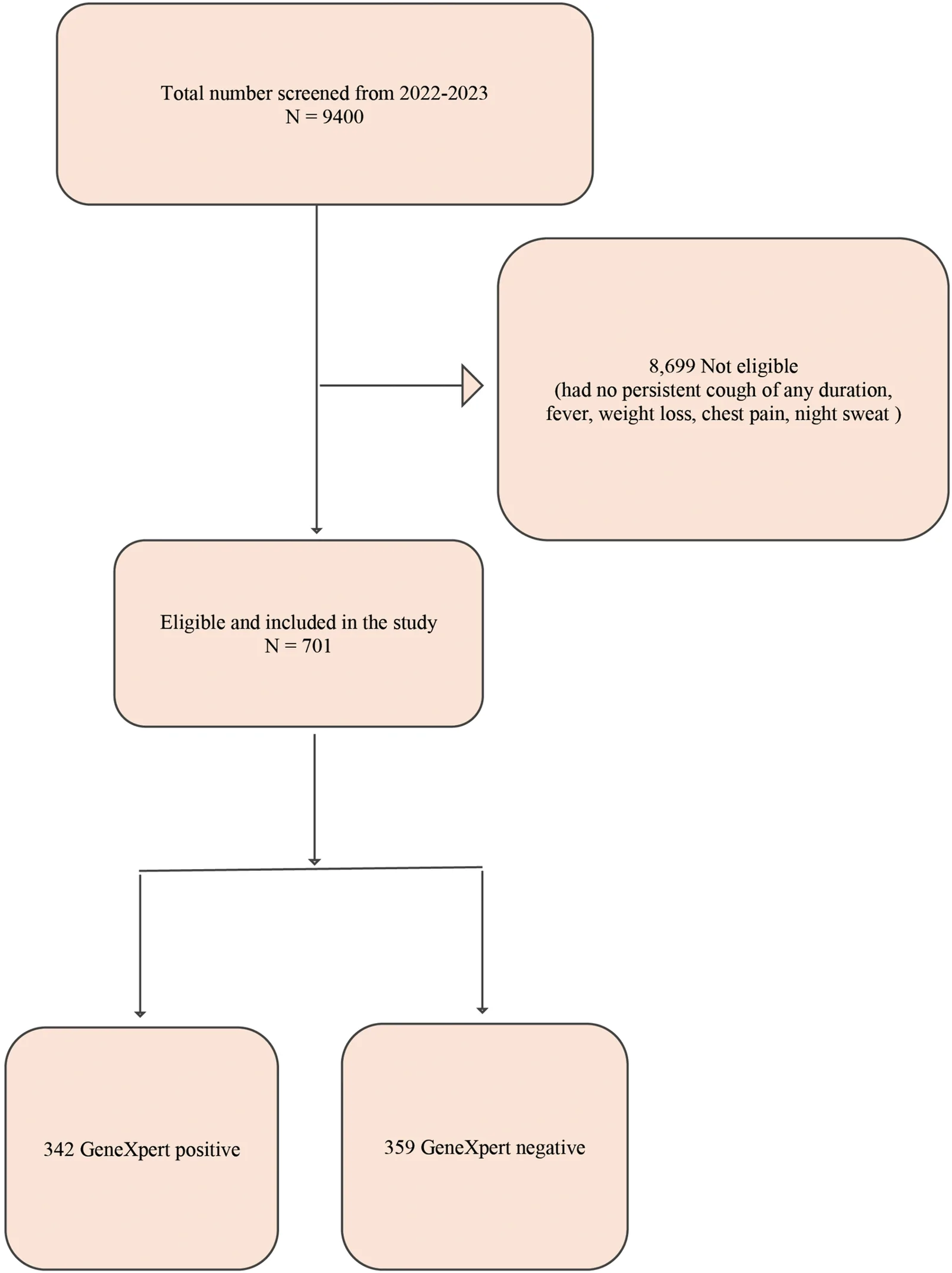
Flowchart of inclusion of persons with Presumptive TB.

The study population comprised persons aged ≥15 years presenting to the Outpatient Department (OPD) from July 2022 to June 2023. Participants were eligible if they presented with a cough of any duration (acute or chronic) or other symptoms suggestive of pulmonary TB (fever, night sweats, weight loss, chest pain) and were capable of producing a sputum sample. The study excluded pregnant women (due to radiation risk), individuals with a history of prior TB treatment in the last 2 years to avoid confounding by post-TB fibrosis. Also patients currently on anti-TB medication were excluded. A total of 9400 were screened at the OPD of the study site to recruit 701 eligble participants (see Fig 1).

### Reference standard and testing process

**Fig 2:**
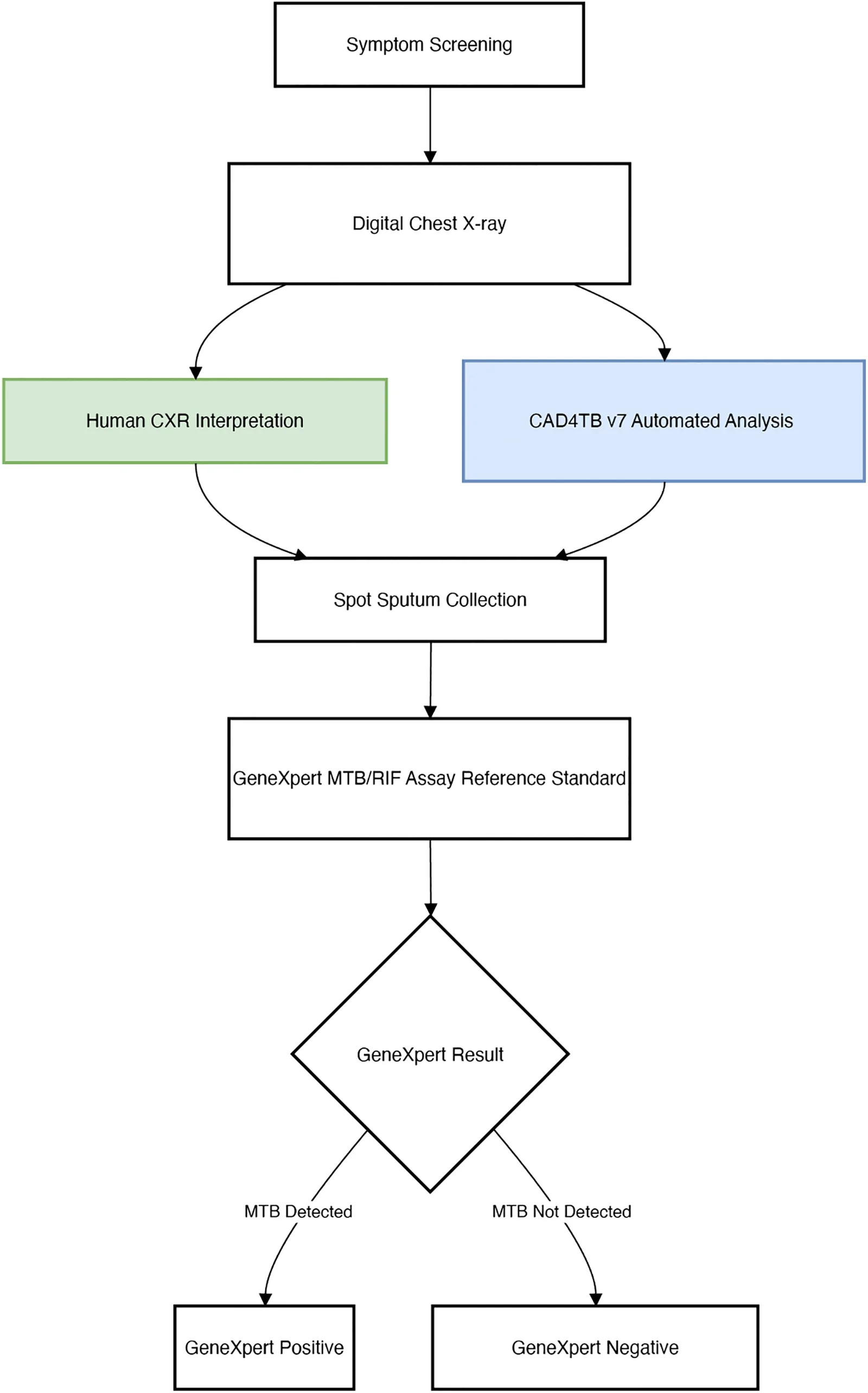
Study Screening and Testing Process.

All eligible participants first underwent symptom screening. Following screening, each participant received a posterior–anterior CXR. The radiographs were interpreted independently by a radiologist and again analyzed using CAD4TB version 7 software. The CAD4TB system applies deep neural network algorithms to evaluate spatial features and texture patterns within the lung fields and generates a continuous abnormality score ranging from 0 to 100 [6,16]. The AI analysis was conducted automatically upon image acquisition and was performed blind to the GeneXpert results. All participants further provided a spot sputum sample which was analyzed using the GeneXpert MTB/RIF assay (Cepheid, Sunnyvale, CA, USA). This WHO-recommended rapid molecular diagnostic test served as the bacteriological reference standard [8]. A GeneXpert-positive was defined as a participant with *Mycobacterium tuberculosis* (MTB) detected (Trace, Very Low, Low, Medium, or High). A GeneXpert-negative was defined as a symptomatic participant with MTB not detected. Health-related quality of life was measured using the EQ-5D-5L questionnaire administered to all participants [17]. With respect to the index test, all participants underwent a posterior-anterior digital chest radiograph. The testing process is summarized in Fig 2.

### Data management and statistical analysis

Raw data were structured into a dataframe consisting of 701 observations and 35 variables. To ensure robust analysis of health outcomes, missing values in health utility dimensions (usual activities, pain/discomfort, and anxiety/depression) were addressed using Multiple Imputation by Chained Equations (MICE). The imputation model utilized age, sex, TB status, illness duration, and CAD4TB scores as predictors to generate a complete dataset for Quality-adjusted life years (QALYs) calculations.

Responses across the five EQ-5D domains (mobility, self-care, usual activities, pain/discomfort, and anxiety/depression) were first recoded into numeric severity levels ranging from 1 (no problems) to 5 (extreme problems). EQ-5D index utility scores were then calculated using a value-set–based additive approach [17]. EQ-5D index utility scores were calculated using a value-set–based additive approach, yielding a continuous health utility index where 1 represents full health and 0 is equivalent to death. The baseline Quality-Adjusted Life Years (QALYs) lost during the current illness episode were calculated for each participant by multiplying their derived disutility (1 - EQ-5D index) by their self-reported illness duration (converted to years). To conduct a true cost-effectiveness analysis that accounts for the clinical consequences of diagnostic errors, a dynamic morbidity penalty was incorporated. For True Negatives and False Positives (who ultimately test negative on GeneXpert and avoid unnecessary TB treatment), baseline QALYs lost were maintained. True Positives (individuals with TB correctly identified by the CAD threshold) also maintained their baseline QALYs lost, assuming timely initiation of treatment. Crucially, False Negatives—individuals with confirmed TB who scored below the CAD triage threshold and were consequently missed by the screening algorithm—were subjected to a severe morbidity penalty. Their baseline QALYs lost were multiplied by a factor of 5.0 to reflect the prolonged duration of suffering and heightened mortality risk associated with undetected and untreated active tuberculosis in the community.

The primary economic outcome was the Incremental Cost-Effectiveness Ratio (ICER), calculated as the difference in total diagnostic costs between the two strategies divided by the difference in total QALYs lost:

$\text{ICER}=\frac{{\text{Cost}}_{\text{New}}{-\text{Cost}}_{\text{R}\text{eference}}}{\left(PerfectHealth-{\text{QALY}}_{\text{New}}\right)-\left(PerfectHealth-{\text{QALY}}_{\text{Reference}}\right)}$

$\text{ICER}=\frac{{\text{Cost}}_{\text{New}}{-\text{Cost}}_{\text{R}\text{eference}}}{{\text{QALY}\text{ Lost}}_{\text{Reference}}-{\text{QALY}\text{ Lost}}_{\text{New}}}$

Because the health outcome evaluated was a burden of disease (QALYs lost), the Incremental Net Monetary Benefit (NMB) was calculated using the formula: NMB = (QALYs_lost avoided × WTP) – Cost. This negative formulation ensures that strategies which maximize NMB are those that successfully minimize both financial costs and patient suffering. The analysis utilized a local willingness-to-pay (WTP) threshold of $1,103 per QALY, representing 0.5 × Ghana's GDP per capita [18].

**Fig 3:**
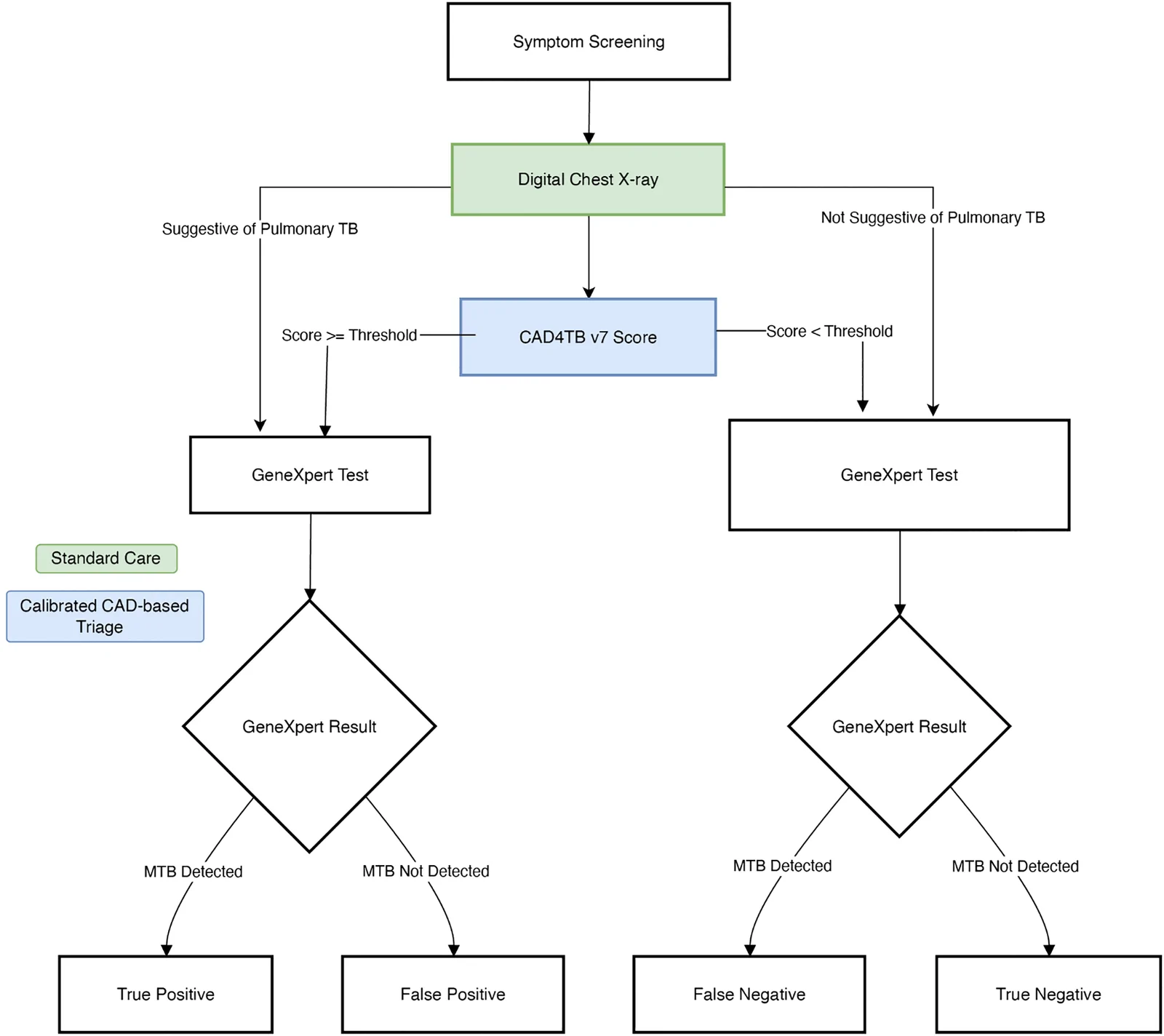
Decision Algorithms.

The analysis compared the cost effectivenes of the vendor threshold of 51, developed based on the manufacturer’s calibration studies and validation analyses [19], against locally calibrated thresholds of 56 (weighted Youden index optimized for WHO-compliant sensitivity ≥90% [20]) and 68 (standard Youden index prioritizing balanced accuracy) (see Fig 3).

These CAD-integrated triage pathways were subsequently evaluated against the current Standard of Care (symptom screening followed by human-read CXR before GeneXpert testing). Costs were estimated in United States Dollars (USD) at an exchange rate of 12 Ghana Cedis (GHS) per 1 USD. The unit costs applied were $1.00 for symptom screening [2], $20.49 for digital CXR, $43.90 for GeneXpert MTB/RIF testing (a comprehensive figure inclusive of cartridges and logistics) [2], and $5.71 for CAD software analysis [4]. All statistical and economic analyses were performed using R statistical software version 4.3

### Ethical considerations

Ethical clearance was obtained from the Committee for Human Research and Publication of the Kwame Nkrumah University of Science and Technology (Ref: CHRPE/AP/269/22) and the Ghana Health Service Ethics Review Committee. Administrative approval was granted by the Director-General of the Ghana Health Service. All participants provided written informed consent. For participants aged 15–17 years, assent was obtained alongside parental/guardian consent.

## Results

### Study population characteristics

A total of 701 symptomatic individuals were included in the study, comprising 342 (48.8%) bacteriologically confirmed GeneXpert-Positives and 359 (51.2%) GeneXpert-Negatives. The baseline socio-demographic and clinical characteristics of the study population are summarized in Table 2. The mean age was comparable between GeneXpert-positives (44.3 years) and GeneXpert-negatives (46.6 years, p = 0.057). Significant demographic disparities were observed; GeneXpert-positives were predominantly male (81.6% vs. 44.6%, p < 0.001) and more likely to be involved in mining (24.9% vs. 8.4%, p < 0.001). Clinically, GeneXpert-positives presented with a significantly higher prevalence of suggestive symptoms, including cough > 2 weeks (82.5%), chest pain (73.1%), and unintentional weight loss (73.1%) compared to GeneXpert-negatives (p < 0.001). Furthermore, smoking history was more prevalent among GeneXpert-positives, with 39.1% being current or past smokers compared to 11.6% of GeneXpert-negatives (p < 0.001). The mean CAD4TB score was significantly higher in the GeneXpert-positives group (81.7 ± 19.7) than in the GeneXpert-negatives group (49.4 ± 24.5, p < 0.001).

**Table 2:** Socio-Demograpahics of Study Participants.

Characteristic	N	GeneXpert-Positive N = 342^*1*^	GeneXpert-Negative N = 359^*1*^	p-value^*2*^
Age (years)	701	44.3 (13.9)	46.6 (16.7)	0.057
Age group (years)	701			<0.001
Child [0–14]		0 (0.0%)	0 (0.0%)	
Young Adult [15–30]		44 (12.9%)	65 (18.1%)	
Middle Aged [31–4 5]		163 (47.7%)	126 (35.1%)	
Adult [46–60]		97 (28.4%)	89 (24.8%)	
Elderly [>60]		38 (11.1%)	79 (22.0%)	
Sex	701			<0.001
Male		279 (81.6%)	160 (44.6%)	
Female		63 (18.4%)	199 (55.4%)	
Occupation category	701			<0.001
Agriculture		50 (14.6%)	45 (12.5%)	
Mining		85 (24.9%)	30 (8.4%)	
Others		44 (12.9%)	90 (25.1%)	
Services/Skilled/Manual		87 (25.4%)	101 (28.1%)	
Students/Unemployed		76 (22.2%)	93 (25.9%)	
Type of residence	701			0.012
Rural		144 (42.1%)	118 (32.9%)	
Urban		198 (57.9%)	241 (67.1%)	
Diabetes status	701	18 (5.3%)	35 (9.7%)	0.025
Hypertension status	701	12 (3.5%)	29 (8.1%)	0.010
Smoking status	701			<0.001
Never smoked		208 (60.8%)	317 (88.3%)	
Currently smoke		47 (13.7%)	21 (5.8%)	
Past smoker		87 (25.4%)	21 (5.8%)	
Cough > 2 weeks	701	282 (82.5%)	252 (70.2%)	<0.001
Chest pain	701	250 (73.1%)	82 (22.8%)	<0.001
Unintentional weight loss	701	250 (73.1%)	58 (16.2%)	<0.001
Night sweats	700	174 (51.0%)	39 (10.9%)	<0.001
Fever	701	212 (62.0%)	85 (23.7%)	<0.001
CAD4TB score	701	81.7 (19.7)	49.4 (24.5)	<0.001

^*1*^Mean (SD); n (%).

^*2*^Wilcoxon rank sum test; Fisher's exact test; Pearson's Chi-squared test.

### Health-related quality of life and morbidity

Health utility outcomes and morbidity burden are presented in S1 Fig. The median EQ-5D health utility index was significantly lower for confirmed GeneXpert-positives (0.8 [0.4–1.0]) compared to GeneXpert-negatives (0.9 [0.8–1.0], p < 0.001). GeneXpert-positives also experienced a significantly longer median duration of illness (7 [1.0, 12.0]months) compared to GeneXpert-negatives (0.1 [0.1, 0.2]months, p < 0.001).

Stratified analysis (Table 3) revealed that males reported significantly lower health utility scores (0.6) than females (0.7, p = 0.012). Additionally, urban residents reported lower utility (0.6) than those in rural areas (0.7, p = 0.026). With QALY lost (Table 3), health utility scores varied significantly by age group (p = 0.043) and residence (p = 0.073).

**Table 3:** Table: Stratified Analysis of Health Utility and Clinical Characteristics.

Characteristic	N		EQ-5D health utility index (Mean ± SD)	p-value	Illness duration, months (Mean ± SD)	p-value	QALY Lost (Mean ± SD)	p-value
**Age group**	342	Young Adult [15–30]	0.7 (0.3)	0.10¹	7.2 (5.1)	0.80¹	0.4 (0.4)	0.043¹
		Middle Aged [31–45]	0.7 (0.4)		6.8 (4.8)		0.3 (0.3)	
		Adult [46–60]	0.7 (0.3)		6.6 (5.2)		0.4 (0.4)	
		Elderly (>60)	0.6 (0.3)		6.3 (5.3)		0.3 (0.3)	
**Sex**	342	Male	0.6 (0.3)	0.012²	6.6 (5.0)	0.30²	0.3 (0.4)	0.20²
		Female	0.7 (0.3)		7.3 (5.2)		0.4 (0.4)	
**Residence**	342	Rural	0.7 (0.3)		6.3 (4.9)		0.3 (0.4)	
		Urban	0.6 (0.4)	0.026²	7.0 (5.1)	0.30²	0.3 (0.3)	0.073²

^1^Kruskal–Wallis rank sum test.

^2^T-test.

### Diagnostic performance, cost-effectiveness of CAD4TBv7 thresholds

The diagnostic performance and isolated cost-effectiveness of the CAD4TB thresholds (vendor-recommended 51, weighted Youden 56, and standard Youden 68) are presented in Table 4. When evaluating the CAD triage step in isolation, the vendor threshold of 51 was the most sensitive (91.5%) and therefore minimized the total QALYs lost (223.64 QALYs) but incurred the highest diagnostic cost of US$23,933.31 due to a high volume of GeneXpert referrals. Increasing the threshold to 56 reduced sensitivity to 90.4% but improved specificity, resulting in diagnostic cost savings of US$1,185.30. However, the missed GeneXpert-positives at this threshold generated an incremental health loss of 1.08 QALYs compared to threshold 51. The ICER of US$1,097.33 per QALY lost was marginally lower than the WTP threshold of US$1,103, resulting in a slightly negative incremental Net Monetary Benefit (NMB) of −US$6.12. Conversely, raising the threshold to the standard Youden index of 68 yielded substantial financial savings (US$2,985.20) but caused a severe drop in sensitivity (85.1%). This led to a significant health penalty of 20.53 incremental QALYs lost. The financial savings did not justify this health loss, resulting in an ICER of US$145.39 per QALY lost (far below the compensation required by the WTP threshold) and a negative incremental NMB of US$19,662.15.

**Table 4:** Diagnostic Performance and Cost-Effectiveness of CAD4TB Thresholds.

Threshold	N Positive	N Negative	Sensitivity	Specificity	QALYs Lost -- GeneXpert-Positive (mean)	QALYs Lost -- GeneXpert-Negatives (mean)	Total QALYs Lost	Total Cost (USD)^*1*^	Incremental Cost	Incremental QALYs	ICER^*2*^	Incremental NMB^*3*^
**51**	454	247	91.5%	0.61	0.36	0.25	223.64	23,933.31	Reference	Reference	Reference	Reference
**56**	427	274	90.4%	0.67	0.38	0.23	224.72	22,748.01	−1,185.30	−1.08	1,097.33	−6.12
**68**	386	315	85.1%	0.74	0.39	0.29	244.17	20,948.11	−2,985.20	−20.53	145.39	−19,662.15

^*1*^Total costs include CAD software for all 701 individuals ($5.71 each) plus GeneXpert confirmatory testing ($43.90) for CAD-positive individuals only.

Symptom screening and CXR costs are excluded as they are common to all threshold comparisons.

^*2*^ICER: Negative ICER indicates cost-saving with reduced QALY burden.

^*3*^Incremental NMB calculated at willingness to pay = $1,103 per QALY(0.5x Ghana GDP per capita).

### Cost-effectiveness of diagnostic pathways

**Fig 4:**
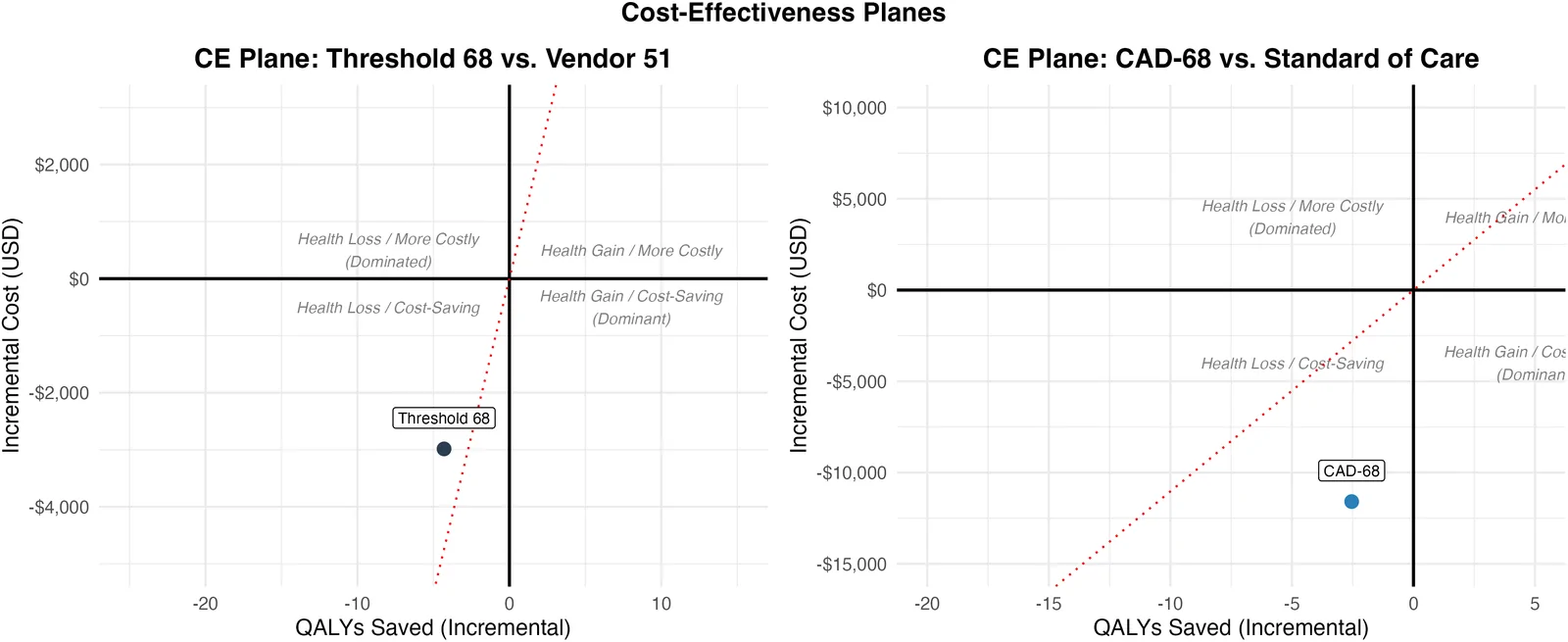
Cost Effectiveness Plane of Thresholds 68.

**Fig 5:**
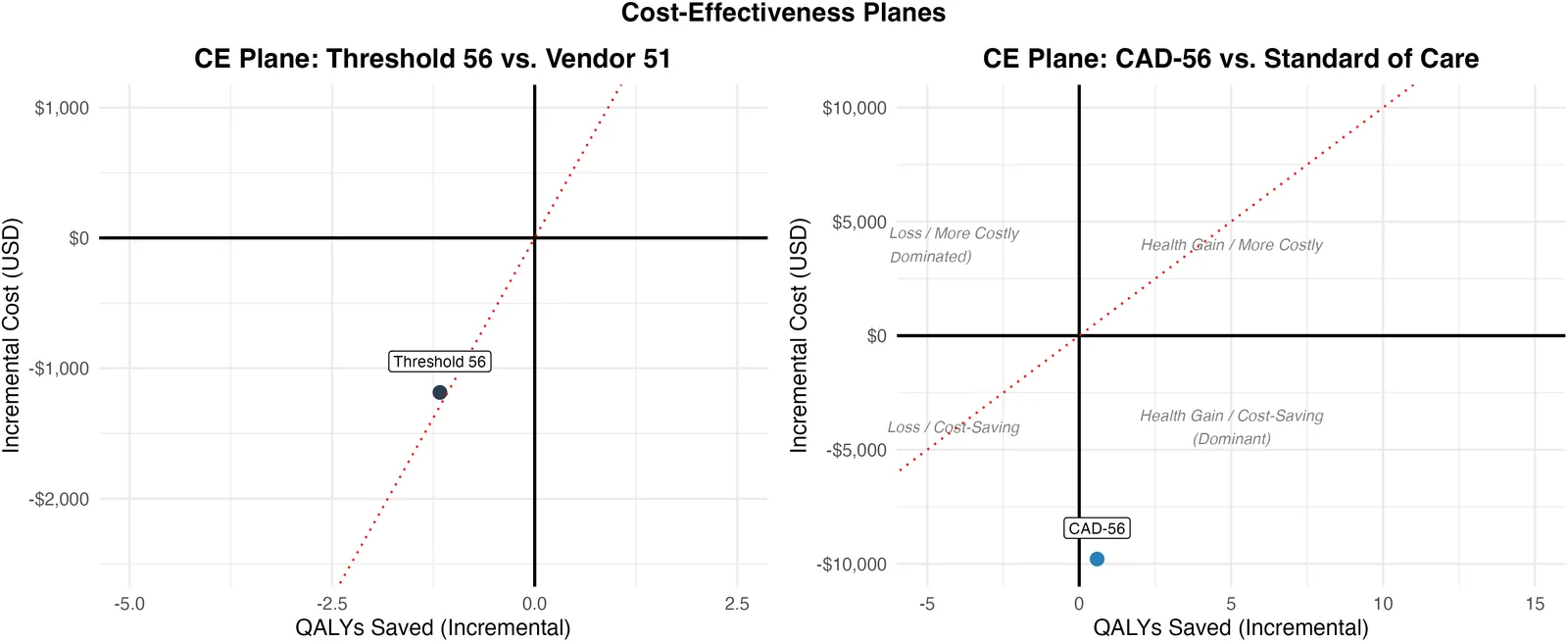
Cost Effectivenes Plane of Thresholds 56.

The cost-effectiveness of the full diagnostic pathways, comparing the Standard of Care (SoC) against the CAD-integrated triage strategies, is presented in Table 5. The SoC incurred the highest total cost of US$33,239.09, whereas the total pathway costs for CAD thresholds of 51, 56, and 68 were substantially lower at US$24,634.31, US$23,449.01, and US$21,649.11, respectively (see Fig 4 and 5). In terms of health outcomes, the CAD strategies with high sensitivity (thresholds 51 and 56) strictly dominated the SoC. Both thresholds 51 and 56 were associated with fewer total QALYs lost than the SoC (223.64 and 224.72 versus 234.80), resulting in absolute health gains of 11.16 and 10.08 QALYs, respectively. These strategies resulted in negative ICERs (−US$771.14 and −US$971.40) and generated substantial incremental net monetary benefits (US$20,912.61 and US$20,906.48) at the local WTP threshold of US$1,103/QALY.

**Table 5:** Cost-Effectiveness of Diagnostic Pathways ^1^ (Standard of Care vs. CAD-integrated triage).

Pathway	Threshold definition	Total QALYs Lost	Cost per pathway	Total Cost (USD)	NMB	Incremental Cost (vs SoC)	Incremental QALYs (vs SoC)	ICER (vs SoC)	Incremental NMB (vs SoC)^*2*^
Pathway A (SoC)	Expert Reading of Xray suggestive of PTB	234.80	80.29	33,239.09	−292,222.02	Reference	Reference	Reference	Reference
Pathway B (CAD-51)	CAD4TB score > 51	223.64	54.26	24,634.31	−271,309.41	−8,604.78	11.16	−771.14	20,912.61
Pathway C (CAD-56)	CAD4TB score > 56	224.72	54.92	23,449.01	−271,315.54	−9,790.08	10.08	−971.40	20,906.48
Pathway D (CAD-68)	CAD4TB score > 68	244.17	56.09	21,649.11	−290,971.56	−11,589.98	−9.37	1,236.40	1,250.46

^*1*^Pathway A = Standard of Care (symptom screening + human-read CXR + GeneXpert).

Pathways B-D = CAD-integrated triage at specified thresholds.

Total cost = n_positive × cost_per_test. Costs in USD.

^*2*^Positive incremental NMB indicates cost-effectiveness relative to Standard of Care at Willingness to pay = $1,103 per QALY.

## Discussion

The implementation of CAD software as a gatekeeper for molecular testing represents a transformative shift in Ghana's pulmonary TB diagnostic landscape. This study aimed to provide the scientific basis on the cost effectiveness of various CAD-based triage strategies against the current standard of care in Ghana to inform a further assessment for sustainable, national-scale rollout of CAD4TB.

### Summary of main findings

The primary finding of this study is that threshold 56 is a dominant economic strategy compared to the current SoC. By replacing human-read CXR with CAD-integrated triage at the optimized threshold of 56, the health system reduces total diagnostic pathway costs by nearly 30% (decreasing from US$33,239.09 to US$23,449.01 for 701 symptomatic individuals). Because CAD-56 achieves a higher sensitivity than the human-read proxy, this financial saving is accompanied by an incremental health gain of 10.08 QALYs. The economic model has demonstrated the danger of prioritizing financial savings over diagnostic sensitivity. When the CAD threshold was raised to 68 (optimizing for balanced accuracy), the health system saved additional funds on GeneXpert cartridges but missed a significant number of true positives. Due to the prolonged morbidity and mortality risks associated with untreated TB, threshold 68 generated a severe health penalty (incremental QALYs of −9.37 compared to SoC) and an ICER that far exceeded the local willingness-to-pay threshold [18]. Consequently, threshold 56 was identified as the optimal balance, maximizing financial sustainability without compromising patient health.

### Health equity and cost-effectiveness

Confirmed GeneXpert-positives had significantly poorer health status and greater morbidity than GeneXpert-negatives. Median EQ-5D health utility was lower among GeneXpert-positives (0.8 [0.4–1.0]) compared with GeneXpert-negatives (0.9 [0.8–1.0], p < 0.001), and GeneXpert-positives reported a substantially longer median duration of illness (7 [1.0–12.0] months vs 0.1 [0.1–0.2] months, p < 0.001). Within the GeneXpert-positives group, males and urban residents experienced significantly worse health utility than females and rural residents, respectively.

The economic value of CAD-integrated triage is primarily driven by the high unit cost of molecular tests. In Ghana, a single GeneXpert test costs approximately US$43.90 per person when accounting for cartridges, personnel, and logistics [2]. Although recent global framework agreements have reduced cartridge prices [21], molecular testing remains a major expenditure for the National TB Control Programme. The analysis confirms that the CAD threshold 56 triage approach sits in the fourth quadrant of the cost-effectiveness plane, denoting strong dominance (more effective and less costly). It generated a higher incremental Net Monetary Benefit (NMB) of US$20,906.48 at a local willingness-to-pay threshold of US$1,103 per QALY [18], strongly supporting its candidature for further study.

### Comparison to other studies

The finding that the CAD threshold 56 is both more effective and less expensive than the standard of care aligns with evaluations in Pakistan and Nigeria. In Pakistan, the cost per screened subject using CAD4TB was approximately half that of screening without AI, while simultaneously achieving 2.5 times higher daily throughput [7]. Similarly, a study in Northeast Nigeria found that an integrated approach using CXR and AI was less expensive and more effective than symptom-only screening when asymptomatic TB prevalence exceeded 30% [22]. The substantial reduction in total health system costs observed in this study mirrors findings by Bashir et al. (2022) [7], who reported that per-screen costs for CAD4TB are heavily reduced compared to human radiologists.

Additionally, the findings regarding the significant morbidity of TB patients (median utility of 0.8 compared to 0.9 for GeneXpert-negatives) are consistent with regional studies. In South Africa, GeneXpert-positives exhibited lower utility scores (0.62) compared to healthy GeneXpert-negatives (0.85) [23]. The disparities observed in this study, where males and urban residents experienced significantly worse health utility, are supported by broader African epidemiological trends [2,22,24].

### Strengths and limitations

A major strength of this study is the application of the Ghana-specific EQ-5D-5L value set to measure utility. This value set, derived from social preferences in Ghana, indicates that mobility and pain/discomfort cause the largest health decrements (0.369 and 0.312 respectively), providing a more culturally relevant estimation of QALYs than international proxies. However, the study has limitations. The CAD accuracy is known to decrease in older populations (≥60 years) and those with a prior history of TB due to residual lung scarring. Furthermore, the static decision-analytic model of this study does not account for the indirect benefits of averted transmission, which are better captured by dynamic models. Finally, the CAD thresholds are not applicable to persons less than 15 years old.

### Strategic policy recommendations

Based on findings of this study, a further national study before national rollout of CAD is recommended as a triage tool for all symptomatic outpatients and high-risk groups in Ghana to validate the CAD threshold of 56. Scaling up should leverage existing digital X-ray infrastructure and integrate with digital health records platforms to reduce diagnostic turnaround times and improve linkage to care. Strategic deployment of mobile digital X-ray units equipped with CAD should be prioritized for mining communities (populated mainly by males) and urban slums (populated mainly by the aged), where TB yield and patient morbidity are exponentially higher than national averages.

## Supporting information

S1 Fig Health Utility and Morbidity Comparison. (TIFF)

## Abbreviation

AI: artificial intelligence
CAD: computer-aided detection
CXR: chest radiograph (X-ray)
EQ-5D-5L: EuroQol 5-dimension 5-level (questionnaire)
ICER: incremental cost-effectiveness ratio
NMB: net monetary benefit
NTP: national tuberculosis control programme
QALY: quality-adjusted life year
TB: tuberculosis
WHO: World Health Organization
